# Preliminary insights into the gut microbiota of patients with rheumatoid arthritis in Vietnam

**DOI:** 10.7717/peerj.20521

**Published:** 2025-12-18

**Authors:** Bich Ngoc Nguyen, Lan Thi Ngoc Nguyen, Dinh Thi My Trinh, Hien Thao Nguyen, Tam Thi Thanh Tran

**Affiliations:** 1 Bach Mai Hospital, Hanoi, Vietnam; 2 Hanoi Medical University, Hanoi, Vietnam; 3 University of Science and Technology of Hanoi, Vietnam Academy of Science and Technology, Hanoi, Vietnam; 4 Hanoi University of Science and Technology, Hanoi, Vietnam

**Keywords:** Rheumatoid arthritis, Gut microbiota, Cohort study, Beta diversity, Biomarkers

## Abstract

In Vietnam, rheumatoid arthritis accounts for more than 20% of all joint diseases, with a growing number of young patients. The disease progresses rapidly, but its exact cause remains not fully understood. Environmental and lifestyle factors, such as smoking, pollution, obesity, gut microbiota, and infections, play a role in rheumatoid arthritis development. The presence of Gram-positive bacteria in the gut might promote the release of toxic metabolites into the bloodstream, which in turn triggers joint inflammation. Therefore, this pilot study aimed to compare the gut microbiota in 22 patients with newly diagnosed rheumatoid arthritis and 20 healthy individuals recruited at the Bach Mai Hospital, Hanoi, Vietnam. To this end, we analyzed fecal samples from all participants by 16S rRNA metagenomic sequencing. The sequencing data analysis did not reveal any significant differences in alpha diversity between patients and healthy controls. Conversely, unweighted and weighted UniFrac distances (beta diversity metrics) allowed distinct clustering between groups. The abundance of the *Lactococcus, Solobacterium, Faecalibaculum,* and* Corynebacterium* genera was increased, and that of *Bacteroides* was decreased in patients with rheumatoid arthritis compared with healthy controls. Moreover, patients exhibited distinct gut microbiota profiles in function of their disease activity scores (DAS28-CRP, DAS-ESR), rheumatoid factor, and anti-citrullinated protein antibody concentrations. Overall, our study contributes to bridging this knowledge gap and provides a foundation for the study of gut microbial signatures of autoimmune disease in Vietnamese patients. It also highlights the potential role of gut microbes in rheumatoid arthritis diagnosis and management in Vietnam.

## Introduction

Rheumatoid arthritis (RA) is a chronic autoimmune disease that causes inflammation and mainly affects joints. According to a systematic analysis by the Global Burden of Disease Study, in 2020, 17.6 million individuals were living with RA worldwide, and this number was expected to increase to 31.7 million by 2025. Overall, RA prevalence was 2.6 higher in women than men. The number of cases recorded in western sub-Saharan Africa, Oceania, and southeast Asia was lower compared to other parts of the world, but this may be explained by limited access to data (Collaborators, 2023). In a study on the prevalence of joint diseases in an urban Vietnamese community, RA concerned 0.28% of the total population and 2.3% of all patients with musculoskeletal disorders, and mostly women (83.3%) (Minh Hoa et al., 2003). RA progresses rapidly leading to joint destruction, but the cause is not fully understood. Genetic factors are involved in RA pathogenesis (Klareskog et al., 2006). However, the concordance rates of RA among monozygotic twins is 15%, indicating that other (environmental, lifestyle) factors also are key contributors (Silman et al., 1993). Indeed, previous studies showed that smoking, female sex, air pollution, obesity, microbes and gut microbiota influence RA development (Pradeepkiran, 2019; Zhao et al., 2022).

Many studies on the association between gut microbiota and diseases in general, and RA in particular, have been carried out. For instance, using 16S sequencing, Scher et al. (2013) found that the abundance of *Prevotella copri* was increased and that of *Bacteroides* was decreased in fecal samples from North American patients with newly diagnosed, untreated RA compared with controls. Similarly, *P. copri* abundance was increased in approximately one-third of the included patients with newly diagnosed RA in Japan (Maeda et al., 2016). A Chinese study reported an enrichment in *Lactobacillus salivarius* and a reduction in *Haemophilus* abundance in the gut and oral microbiota of patients with RA (Zhang et al., 2015). Liu et al. (2013) found that the *Lactobacillus* diversity in feces was higher in patients with RA than in the control group. A study in the USA detected an enrichment of *Eggerthella lenta* and *Collinsella*, two rare lineages, in fecal samples from patients with RA. These findings suggested a link between RA and dysbiosis due to the disease condition, rather than diet or genetic factors (Chen et al., 2016). Conversely, works on the gut microbiota and RA in developing countries, including Vietnam, are lacking. Therefore, the aim of this pilot study was to carry out a metagenomic analysis of 16S sequencing data to investigate and compare the gut microbiota of patients with RA and of healthy individuals in Vietnam. Ultimately, the implementation of microbiome studies in Vietnam will bring useful insights for RA prevention and treatment, and will specifically identify candidate biomarkers that can be used for RA diagnosis/monitoring in Vietnamese patients.

## Materials & Methods

### Participant recruitment and data collection

Twenty-two patients with newly diagnosed RA and 20 healthy controls were recruited at Bach Mai Hospital, Hanoi, Vietnam. The participants’ age ranged between 30 and 70 years and all had a body mass index (BMI) within the normal range (18.5–24.9). Exclusion criteria were: history of gastrointestinal disorders, inflammatory bowel disease, or other gut-related conditions such as colorectal cancer, and being on restricted diets including vegetarian, calorie-restricted diets, low-salt or low-sugar, and gluten-free diets. Individuals who had taken antibiotics or proton pump inhibitors within four weeks before recruitment also were excluded. Data on the participants’ age, sex, BMI, blood pressure, depression, medication use, and smoking status were recorded using a screening questionnaire. Additionally, the participants’ family history of inflammatory and digestive disorders was recorded. For patients with RA, additional data were extracted from their medical records, including biochemical markers, blood counts, antibody testing results, Disease Activity Score 28 based on C-reactive protein (DAS28-CRP), Disease Activity Score 28 based on erythrocyte sedimentation rate (DAS28-ESR). DAS28-CRP and DAS28-ESR were used to classify patients with RA in three subgroups according to the European League Against Rheumatism guidelines: high disease activity (score > 5.1), moderate disease activity (3.2–5.1), and remission/low disease activity (≤3.2) (Fransen & Van Riel, 2009; Wells et al., 2009). All participants were instructed to self-collect fecal samples at the hospital or at home. For RA patients, samples were collected prior to any treatment initiation. Home-collected samples were stored at −4 °C and delivered to the laboratory within 8 h. Fresh stool samples were brought to the laboratory on dry ice, aliquoted, and stored at −80 °C.

### Total genomic DNA extraction and 16S rRNA sequencing

Total genomic DNA was extracted from the fecal samples with the QIAamp^®^ PowerFecal^®^ Pro DNA Kit (QIAGEN, Germany) following the manufacturer’s instructions. The protocol included a bead-beating step using a TissueLyser II (QIAGEN Inc, US) and elution with 100 µl of 10 mM Tris buffer solution. The genomic DNA quality and concentration were estimated with a Nanodrop 2000 spectrophotometer (Thermo Fisher Scientific, USA). The extracted DNA was kept at −20 °C until sequencing using a 250PE Illumina MiSeq platform, as described in Nhung et al. (2024).

### Bioinformatics analysis

Raw sequencing data were analyzed using Quantitative Insights into Microbial Ecology (QIIME) 2 v.2024.5 (Bolyen et al., 2019). Forward and reverse primers were trimmed with cutadapt v.4.9. Trimmed sequences were processed with DADA2-plugin in QIIME2 for denoising, dereplication, and chimera filtering. Taxonomic assignment of the representative sequences of amplicon sequence variants (ASVs) was performed by aligning them to the full-length 16S rRNA sequences from the SILVA v138 reference database using the QIIME2 feature-classifier classify-sklearn. Taxa were assigned from phylum to genus level using a default confidence threshold of 70%. After filtering unassigned and Eukaryota taxa, a phylogenetic tree was built for the representative sequences with QIIME2 phylogeny align-to-tree-mafft-fasttree. Rarefaction curves for gut microbiota samples were plotted from the ASV table using vegan’s rarecurve function. Alpha and beta diversity were estimated using the rarefied ASV table obtained at a sequencing depth of 65,756, which corresponded to the lowest read count across all samples, and QIIME2 diversity core-metrics-phylogenetic.

### Statistical analyses

Statistical analyses were carried out with R v4.2.1. Descriptive data were presented as mean and standard deviation (SD) or as median with interquartile range (IQR). The relative abundance of each taxon within a sample was calculated by dividing its absolute count by the total absolute abundance of all taxa in that sample. Significant differences in clinical measures, alpha diversity indices, and relative abundances of bacterial taxa between groups were assessed with the Mann–Whitney *U*-test. The Kruskal–Wallis test was used to compare three groups, followed by the Dunn’s test for subgroups. ANCOM-BC2 (Lin & Peddada, 2024) was applied to absolute abundance data to identify taxa that were differentially abundant between the RA and control groups. Low-abundance taxa were excluded from statistical analyses when detected in fewer than 50% of samples across all study groups. Categorical variables were compared between groups with the Fisher’s exact test. The Benjamini–Hochberg procedure was applied to adjust *p*-values for multiple comparisons. Beta diversity distances were visualized using Principal coordinates analysis (PCoA), and differences among groups were determined with permutational multivariate analysis of variance (PERMANOVA) with 999 permutations.

## Results

### Participants’ clinical characteristics

The study included 22 patients with RA and 20 healthy individuals (54.5% of women in the RA group and 55% in the control group; Fisher’s exact test, *p* = 1). The median age (IQR) was 53.5 (44.3–60.5) years in the RA group and 51 (45–56) years in the healthy control group (Mann–Whitney *U* test, *p* = 0.64). In both groups, BMI was within the normal range (18.5 to 24.9 kg/m^2^). Patients with RA had high disease activity as indicated by their median DAS28-CRP and DAS28-ESR scores: 5.4 (IQR: 4.2–6.2) and 6.2 (IQR: 5.4–6.7), respectively. These values were above the normal threshold of 2.6. Additionally, the concentrations of rheumatoid factor (RF) and anti-citrullinated protein antibodies (ACPA) were increased in 20/22 (90.9%) and 15/22 (68.2%) patients with RA, and some values exceeded the limit of quantification (Table 1).

**Table 1:** Characteristics of the 22 patients with rheumatoid arthritis.

**Characteristics**	**Median**	**Interquartile range**	**Normal range**
Age at diagnosis, years	53.5	44.2–60.5	
Body mass index, kg/m^2^	21.3	19.7–22.2	18.5–24.9
Tender joints (28 joints examined)	11	6.2–15	0
Swollen joints (28 joints examined)	6.5	3–12	0
C-reactive protein, mmol/l	10.9	1.1–30.6	<5
Erythrocyte sedimentation rate, mm/hour	50	14.8–66.8	<10
DAS28-CRP	5.4	4.2–6.2	<2.6
DAS28-ESR	6.2	5.4–6.7	<2.6
Rheumatoid factor, IU/mL	81.6	42.3–116	<14
Anti-citrullinated protein antibodies, U/mL	61	2.6–199.4	<5
Visual analog scale score, mm	60	50–70	<10
Simple disease activity index	32.3	23.4–44.2	<3.3
Clinical disease activity index	30.5	22–41.2	<2.8
Hemoglobin, g/L	131.5	123–136	120–155
White blood cells, ×10^9^/L	9.1	6.4–10.2	4–10
Platelet count, ×10^9^/L	311	254.5–374.2	150–400

### Gut microbial diversity in patients with RA

**Figure 1:**
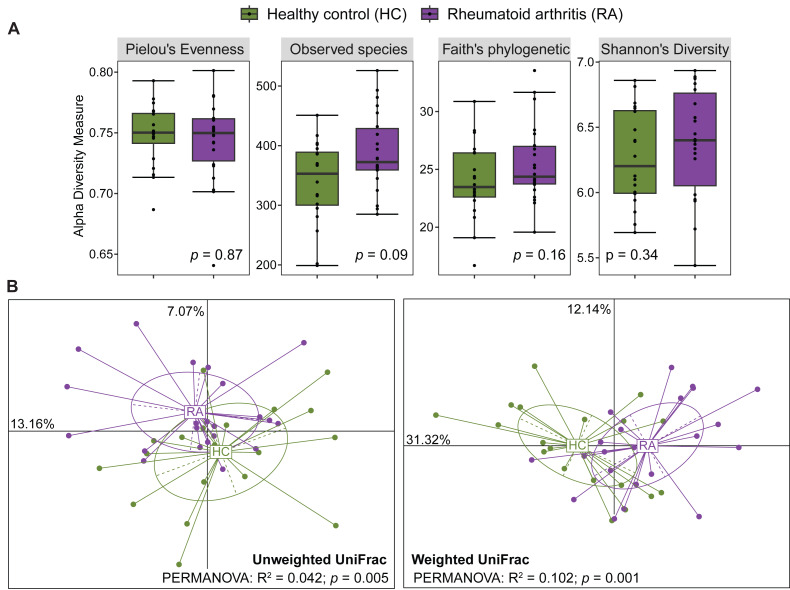
Comparison of alpha diversity and beta diversity between patients with rheumatoid arthritis and healthy controls. Comparison of alpha diversity (A) and beta diversity (B) between patients with rheumatoid arthritis and healthy controls. Differences were estimated using the Mann–Whitney *U* test (alpha diversity) and PERMANOVA (beta diversity).

After processing with QIIME2, the number of reads across the 40 samples ranged from 65,756 to 113,385. The rarefaction curves of all samples reached a plateau above 40,000 reads (Fig. S1), indicating that the ASV table rarefied to 65,756 reads was sufficient for calculating both alpha and beta diversity. The gut microbiota alpha diversity was assessed by calculating the species evenness (Pielou’s evenness, Shannon’s diversity) and species richness (Observed species, Faith’s phylogenetic diversity). All these alpha diversity measures were not significantly different between patients with RA and healthy controls (Mann–Whitney *U* test, *p* > 0.05) (Fig. 1A). Beta diversity was evaluated by computing the unweighted and weighted UniFrac distances that measure the presence/absence of bacterial taxa and their abundance, respectively. Samples from patients with RA and healthy controls could be clustered in two groups using the unweighted UniFrac distances (PERMANOVA: *R*^2^ = 0.042, *p* = 0.005) and also the weighted UniFrac distances (PERMANOVA: *R*^2^ = 0.102, *p* = 0.001) (Fig. 1B).

**Figure 2:**
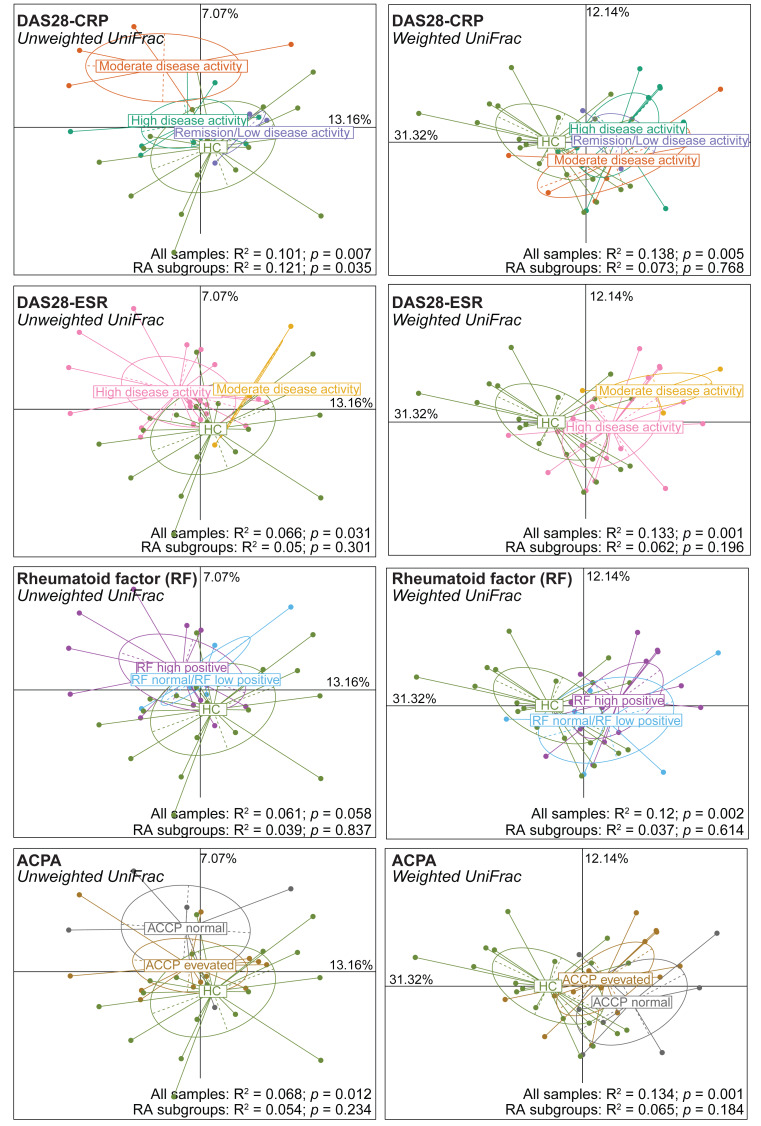
Beta diversity (Unweighted and Weighted UniFrac distance metrics) in and healthy controls (HC) and patients with rheumatoid arthritis (RA) grouped using clinical indicators. Patients were classified in different subgroups as described in Fig. S2. Significant differences between RA subgroups and HC, as well as among RA subgroups, were assessed using PERMANOVA.

Next, alpha and beta diversity were investigated in patients with RA divided in different disease activity groups based on their DAS28-CRP and DAS28-ESR scores, as well as RF and ACPA concentrations. Species evenness and richness were not different across disease activity categories (DAS28-CRP and DAS28-ESR scores) (Figs. S2A and S2B). Conversely, when patients were divided based on the RF and ACPA concentrations in blood, the Shannon’s diversity index was increased in patients with high RF, defined as a concentration more than three times the normal value (>42 IU/mL), compared with patients with normal or low RF concentration (Mann–Whitney *U* test, *p* = 0.049) (Fig. S2C). Similarly, Pielou’s evenness was higher in patients with elevated ACPA concentration (>5 U/mL) than in those with low ACPA levels (Mann–Whitney *U* test, *p* = 0.032) (Fig. S2D). Beta diversity was similar in the different patient subgroups based on the DAS28-ESR, RF and ACPA values, except for the unweighted UniFrac distance that was different in the two disease activity subgroups defined by DAS28-CRP (PERMANOVA: *R*^2^ = 0.121, *p* = 0.035). When RA subgroups based on DAS28-CRP were plotted together with healthy controls using unweighted UniFrac distance, the microbiota profiles of healthy individuals clustered more closely with those in remission/low and high disease activity, while remaining distant from the moderate disease activity group. Furthermore, no clear transitional trajectory from healthy individuals to low, mediate and high disease activity was observed for other clinical indicators in either unweighted or weighted UniFrac distance (Fig. 2). These findings suggest that microbiota shifts do not consistently align with disease activity.

### Gut microbiota composition in patients with RA and healthy controls

**Figure 3:**
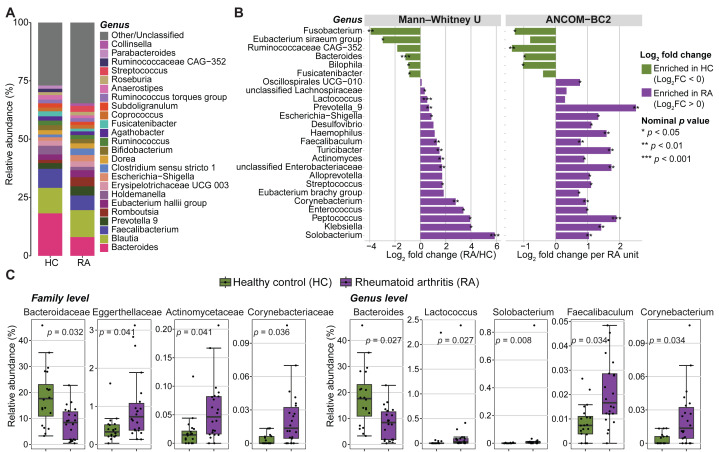
Abundance of gut microbiota components in patients with rheumatoid arthritis (RA) and healthy controls (HC). (A) Mean relative abundances of bacterial genera in the RA and HC groups. (B) Genera with significantly different abundance in the RA and HC groups were identified using either the Mann–Whitney *U* test or ANCOM-BC2 (nominal *p* values < 0.05). Violet, taxa enriched in the RA group; green, taxa enriched in the HC group. (C) Bar plots showing the relative abundance of families and genera that remained significantly different between groups after Mann–Whitney *U* test *p* values were adjusted for multiple comparisons using the Benjamini–Hochberg method. Adjusted *p* values are presented in the bar plots.

Next, the microbial community composition was characterized at different taxonomic levels. The most prevalent bacterial phyla in both groups (RA and controls) were Firmicutes (69.65% ± 11.07%), Bacteroidota (20.62% ± 9.48%), Proteobacteria (4.82% ± 6.73%), and Actinobacteria (4.05% ± 4.60%). They represented >50% of the taxa in most samples. The microbiota composition displayed high variability among individuals in both groups at both the family and genus levels (Fig. S3). The five most frequent families across individual samples were *Lachnospiraceae, Ruminococcaceae, Bacteroidaceae, Prevotellaceae* and *Enterobacteriaceae* (Fig. S3A), while *Bacteroides, Blautia, Faecalibacterium, Prevotella 9* and *Romboutsia* were the most common genera (Fig. 3A and Fig. S3B).

The relative abundance of three phyla, eleven families, and eighteen genera was significantly different between RA and control groups (nominal *p* values <0.05, Mann–Whitney *U* tests) (Fig. 3B, Fig. S4 and Table S1). Similarly, significant differences in absolute abundance were detected for one phylum, twelve families, and twenty-one genera between groups (nominal *p* values <0.05, ANCOM-BC2) (Fig. 3B, Fig. S4 and Table S2). Of these, one phylum, nine families, and fifteen genera were consistently identified by both the Mann–Whitney *U* test and ANCOM-BC2, supporting the robustness of these findings. After correction of Mann–Whitney *U* test *p* values for multiple comparisons, only the abundance of two phyla, four families and five genera remained significantly different between groups (Fig. 3C and Table S1). Specifically, the *Bacteroidaceae* family was enriched in healthy controls compared with patients with RA (BH-adjusted *p* value = 0.032). Conversely, *Actinomycetaceae, Eggerthellaceae* and *Corynebacteriaceae* were more abundant in patients with RA (BH-adjusted *p*-value <0.05). *Lactococcus*, *Solobacterium, Faecalibaculum* and *Corynebacterium* also were enriched in the RA group, whereas *Bacteroides* was decreased (BH adjusted *p*-value <0.05). No phylum, family, or genus remained significant in ANCOM-BC2 after adjusting for multiple comparisons. However, at the ASV level, six ASVs including ASV_910 (g_*Bacteroides*), ASV_1695 (f_*Enterobacteriaceae*), ASV_2473 (g_*Alistipes*), ASV_3334 (g_*Klebsiella*), ASV_1850 (g_*Alistipes*), and ASV_3634 (g_*Christensenellaceae* R-7 group) remained significant after adjustment (BH-adjusted *p*-value <0.05) (Table S2).

### Relationship between gut microbial profiles and clinical indicators of RA

**Figure 4:**
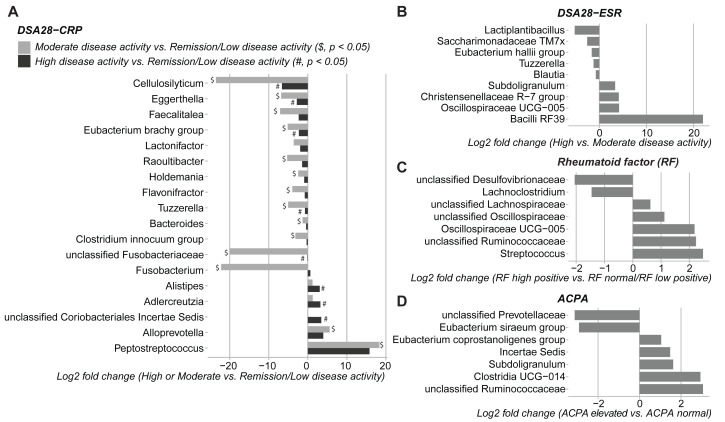
Differences in gut microbiota composition (genera) in patients with rheumatoid arthritis classified in different subgroups of disease activity/severity. (A) Moderate and high disease activity *versus* remission/low disease activity, based on their DAS28-CRP score; (B) high *versus* moderate disease activity, based on their DAS28-ESR score; (C) high *versus* normal/low rheumatoid factor concentration; and (D) elevated *versus* normal anti-citrullinated protein antibody (ACPA) concentration. Only genera with *p* values < 0.05 (Dunn’s test for the DAS28-CRP subgroups and Mann–Whitney *U* test for the other subgroups) are shown; data are presented as log_2_ fold changes. $ and #, significant differences between the moderate or high disease activity DAS28-CRP subgroup and the remission/low disease activity DAS28-CRP subgroup, respectively.

**Figure 5:**
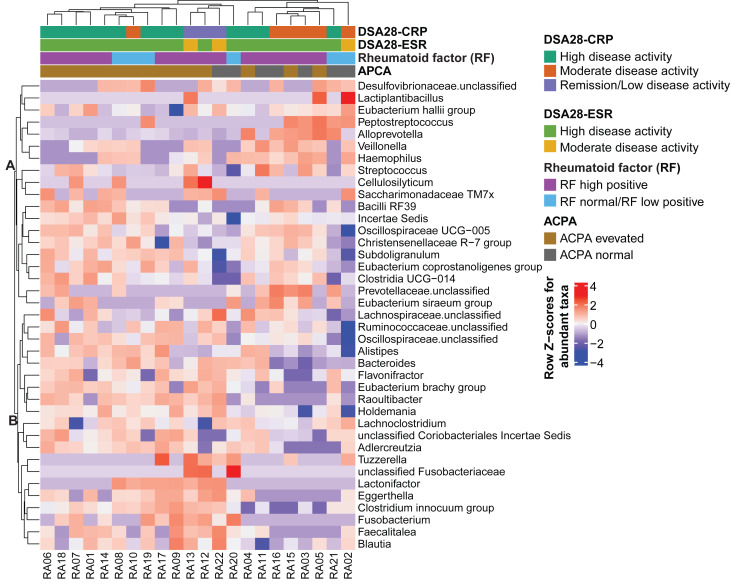
Heatmap of genera the relative abundance of which was significantly different among the indicated subgroups of patients with rheumatoid arthritis. Genera and samples were clustered using Euclidean distances. Data (normalized values) are shown as row *Z*-scores of the relative abundances (see color code). The color bars above the heatmap indicate the RA subgroups based on DAS28-CRP, DAS28-ESR, rheumatoid factor, and anti-citrullinated protein antibodies (ACPA) levels.

Then, the gut microbiota composition was compared across clinical categories defined by the DAS28-CRP and DAS28-ESR scores and RF and ACPA concentrations, as before. The abundance of five families and twenty genera showed significant differences in the high disease activity, moderate disease activity, and remission/low disease activity DAS28-CRP subgroups. Similarly, the abundance of four families and nine genera differed significantly between the high and moderate disease activity DAS28-ESR subgroups. Only *Tuzzerella* abundance was decreased in both high and moderate disease activity subgroups compared with the remission/low subgroup (DAS28-CRP), and also in the high *versus* moderate DAS28-ESR subgroup (Table S3, Figs. 4A–4B). Moreover, the abundance of seven genera varied between the high RF and low RF subgroups. In the SILVA database, most of these genera were labeled as unclassified, except *Streptococcus* that was increased in the high RF subgroup, and *Lachnoclostridium* that was decreased in the same subgroup (Table S3, and Fig. 4C). The abundance of two genera was decreased in the normal ACPA subgroup, and the abundance of five genera was increased in the elevated ACPA subgroup. Notably, *Subdoligranulum* was positively associated with ACPA concentration (Table S4, and Fig. 4D). To explore the overall patterns, all genera associated with these clinical characteristics were listed to generate a heatmap of their relative abundance. Overall, samples tended to cluster according to the disease activity level; however, some mixing between subgroups remained. Genera could be separated into two clusters (Fig. 5). Cluster A included 19 genera predominantly found in samples from patients with normal ACPA concentration, while Cluster B comprised 20 genera that were more abundant in samples from patients with elevated ACPA concentration (Fig. 5). Although these genera lost statistical significance after correction for multiple testing (Table S3), the link between gut microbiota profiles and RA clinical subgroups should be investigated in larger cohorts.

## Discussion

This is the first study to characterize the fecal microbiota profiles of Vietnamese patients with RA using 16S rRNA sequencing. We compared the gut microbiota diversity and composition in patients with RA and in healthy individuals of similar age, sex and BMI and then in patients with RA classified in different disease activity/severity subgroups (DAS28-CRP, DAS28-ESR, RF and ACPA).

We observed no significant difference in alpha diversity metrics (Pielou’s evenness, Shannon’s diversity, Observed species, and Faith’s phylogenetic diversity) between patients with RA and healthy individuals. This is in agreement with the study by Chen et al. (2021) who analyzed 29 patients with RA and 30 healthy controls in South China and found no significant differences in the Abundance-based Coverage Estimator (ACE), Chao1, Shannon, and Simpson indices using 16S rRNA sequencing and metagenomic approaches. Similarly, in Japan, Kishikawa et al. (2020) reported no significant difference in the Shannon index between 82 patients with RA and 42 healthy controls using shotgun metagenomic analysis. Conversely, two Chinese studies based on 16S rRNA sequencing reported a reduction in species richness in patients with RA compared to healthy controls (Chen et al., 2016; Chen et al., 2021). These inconsistencies may be attributed to differences in study cohorts, study locations, lifestyle and dietary habits, and methodological approaches.

Conversely, beta diversity allowed clustering patients with RA and healthy individuals, like in most published studies (Chen et al., 2016; Sun et al., 2019). Indeed, our analysis identified significant differences in several bacterial lineages in the two groups. For instance, the abundance of the families *Actinomycetaceae* (specifically the *Actinomyces* genus), *Eggerthellaceae* and *Corynebacteriaceae* (specifically the *Corynebacterium* genus) was increased in patients with RA. Higher abundances of *Eggerthella* and *Actinomyces* in patients with RA were previously reported by Chen et al. (2016). *E. lenta*, a species belonging to the *Eggerthellaceae* family, has been implicated in the induction of arthritis in a mouse model of collagen-induced arthritis (Balakrishnan et al., 2023). In this model, *E. lenta* exacerbated arthritis severity by enhancing the immune response to type II collagen, which led to increased levels of pro-inflammatory cytokines in the serum, including interleukin (IL)-9, IL-17, IL-23, interferon (IFN)-γ and tumor necrosis factor (TNF)-α. Additionally, *E. lenta* treatment reduced the number of regulatory T cells (FoxP3^+^) and increased follicular helper T cells (CD4^+^CXCR5^+^) as well as CD4^+^ T cells that secrete IL-17 and IFN-γ. *Actinomyces* are part of the healthy core microbiota (Kononen & Wade, 2015); however, in patients with sporadic early-onset colorectal cancer, the co-presence of this genus with several pro-tumor microbial taxa promotes inflammation through activation of the toll-like receptor (TLR)2/NF-κB and TLR4/NF-κB signaling pathways, and also impair the anti-tumor immune responses by reducing the accumulation of CD8^+^ T lymphocytes (Xu et al., 2022). The mechanism linking the *Actinomyces* genus to RA has not been fully elucidated, but similar to its role in colorectal cancer, it could trigger signaling pathways involved in RA development/progression. Unlike *Actinomycetaceae* and *Eggerthellaceae*, the increased abundance of *Corynebacteriaceae* and its genus *Corynebacterium* has never been reported in patients with RA. Several *Corynebacterium* species, such as *Corynebacterium tuberculostearicum*, *Corynebacterium jeikeium* and *Corynebacterium accolens,* are considered proinflammatory bacteria that can induce inflammatory responses in human skin cells (Altonsy et al., 2020; Ridaura et al., 2018). The abundance of the *Corynebacterium* genus was also increased in the saliva of patients with gastric cancer and may serve as a marker of immune deficiency in these patients (Huang et al., 2021). Thus, the presence of *Corynebacterium* with other pathogenic taxa might significantly contribute to RA progression.

Moreover, *Lactococcus, Solobacterium* and *Faecalibaculum* were positively associated with the RA group. A Norwegian cross-sectional study on the oral microbiota found an association between *Solobacterium*, a genus linked to chronic inflammation, and juvenile idiopathic arthritis (Frid et al., 2020). *Lactococcus*, a member of the lactic acid bacteria group, has probiotic properties and is widely recognized as a beneficial microbe (Hamdaoui et al., 2024). Liu et al. (2013) reported an increased abundance of *Lactobacillus*, a sister genus of *Lactococcus*, in patients with early RA compared with healthy individuals. They emphasized that different *Lactobacillus* species may play a protective or pathogenic role in the context of arthritis. Indeed, *Lactobacillus bifidus* can trigger arthritis in a murine model by disrupting the balance between regulatory T and helper T17 cells and by activating the TLR2–TLR4 signaling pathways (Scher & Abramson, 2011). *Faecalibaculum* is a bacterium with anti-inflammatory properties. Preclinical and clinical studies have demonstrated the probiotic potential of several *Faecalibaculum* species in various diseases, such as inflammatory bowel disease, type 2 diabetes, colorectal cancer, and RA (Martin et al., 2023; Moon et al., 2023). In the present study, the higher abundance of *Faecalibaculum* in the RA group could be due to some *Faecalibaculum* species with negative effects on the disease. More studies using alternative approaches, such as shotgun metagenomics, are necessary to precisely identify the species that contribute to RA development.

Among the genera that were decreased in patients with RA, *Bacteroides* was the only one that remained significant after controlling for the false discovery rate. This is line with the study by Wang et al. (2022). *Bacteroides* species are considered beneficial bacteria for gut health because they can degrade polysaccharides into short-chain fatty acids that are implicated in maintaining the gut barrier and have anti-inflammatory activity (Tufail & Schmitz, 2025). *Bacteroides fragilis*, a species belonging to the *Bacteroides* genus, plays a role in enhancing the host-microbe balance by activating the TLR pathway in T lymphocytes (Round et al., 2011). *B. fragilis* also participates in the modulation of metabolite levels, which affects the therapeutic efficacy of methotrexate in patients with RA (Zhou et al., 2022).

Besides the differences between the RA and control groups, we detected gut microbiota variations in patients with RA classified in different clinical subgroups based on their DAS28-CRP, DAS28-ESR, RF and ACPA values. The association of clinical pathological features with gut microbiota has been previously described (Picchianti-Diamanti et al., 2018; Rooney et al., 2024). Specifically, a reduction in *Prevotellaceae* strains has been associated with multiple factors linked to higher risk of arthritis development (Rooney et al., 2024). Consistently, we observed that the unclassified *Prevotellaceae* genus was decreased in patients with elevated ACPA levels. Other genera, such as *Tuzzerella*, exhibited a negative correlation with high disease activity (DAS28-CRP and DAS28-ESR). A previous study showed that low *Tuzzerella* abundance correlated with downregulation of various metabolic pathways related to glutamatergic synapse signaling, nicotinate and nicotinamide metabolism, and aldosterone synthesis and secretion (Chen et al., 2024). *Streptococcus* abundance was increased in patients with high RF concentration and was also enriched in patients with RA before multiple testing correction. Previous studies reported *Streptococcus* enrichment in the oral and gut microbiota of patients with RA (Lee et al., 2022; Moentadj et al., 2021). *Streptococcus* cell wall contains peptidoglycan-polysaccharide polymers that can trigger arthritis in mice (Moentadj et al., 2021). Moreover, *Streptococcus pyogenes,* a member of the *Streptococcus* genus, induces cytokine production, including TNF and type I IFN, and promotes leukocyte recruitment, phagocytosis, as well as the formation of neutrophil extracellular traps (Fieber & Kovarik, 2014). These findings underscore the potential of gut microbes as informative biomarkers of RA progression and prognosis.

The study has several limitations. First, the use of 16S rRNA amplicon sequencing restricted taxonomic resolution to the genus level and lacked functional information on microbes, limiting the ability to infer whether observed microbial alterations reflect protective, compensatory, or pathogenic mechanisms. Second, the small sample size reduced statistical power and generalizability, particularly for RA subgroup analyses, given the high interindividual variability of the gut microbiota. Third, the associations between microbial features and clinical parameters are descriptive, leaving it unclear whether alterations in the gut microbiota are a cause or a consequence of RA-related clinical changes. Future studies should be conducted in larger cohorts from diverse geographic regions of Vietnam, integrate species-level taxonomic profiling with functional inference, and account for potential confounders such as age, gender, body mass index, region, diet, and lifestyle in the analysis.

## Conclusions

This study contributes to our understanding of the role of gut microbes in RA pathogenesis in Vietnamese patients. Although alpha diversity did not differ between patients with RA and healthy controls, distinct microbial profiles were detected between groups (unweighted and weighted UniFrac distances). Our findings also revealed an increase in some genera in patients with RA, such as *Lactococcus*, *Solobacterium*, *Faecalibaculum* and *Corynebacterium*, whereas *Bacteroides* was decreased. These gut microbiota alterations may contribute to the inflammatory processes involved in RA. Given the potential link between gut microbiota and RA, these findings serve as a foundation for future research into the application of gut microbiota components as biomarkers for RA diagnosis and treatment monitoring in Vietnam.

## Supplemental Information

10.7717/peerj.20521/supp-1 Supplemental Information 1 Supplementary figures

10.7717/peerj.20521/supp-2 Supplemental Information 2 Supplementary tables

## Additional Information and Declarations

### Data Availability

The following information was supplied regarding data availability: The raw sequencing data of this study are available at NCBI BioProject: PRJNA1295194.

## References

[ref-1] Altonsy MO, Kurwa HA, Lauzon GJ, Amrein M, Gerber AN, Almishri W, Mydlarski PR (2020). *Corynebacterium tuberculostearicum*, a human skin colonizer, induces the canonical nuclear factor-kappaB inflammatory signaling pathway in human skin cells. Immunity, Inflammation and Disease.

[ref-2] Balakrishnan B, Luckey D, Wright K, Davis JM, Chen J, Taneja V (2023). Eggerthella lenta augments preclinical autoantibody production and metabolic shift mimicking senescence in arthritis. Science Advances.

[ref-3] Bolyen E, Rideout JR, Dillon MR, Bokulich NA, Abnet CC, Al-Ghalith GA, Alexander H, Alm EJ, Arumugam M, Asnicar F, Bai Y, Bisanz JE, Bittinger K, Brejnrod A, Brislawn CJ, Brown CT, Callahan BJ, Caraballo-Rodríguez AM, Chase J, Cope EK, Da Silva R, Diener C, Dorrestein PC, Douglas GM, Durall DM, Duvallet C, Edwardson CF, Ernst M, Estaki M, Fouquier J, Gauglitz JM, Gibbons SM, Gibson DL, Gonzalez A, Gorlick K, Guo J, Hillmann B, Holmes S, Holste H, Huttenhower C, Huttley GA, Janssen S, Jarmusch AK, Jiang L, Kaehler BD, Kang KB, Keefe CR, Keim P, Kelley ST, Knights D, Koester I, Kosciolek T, Kreps J, Langille MGI, Lee J, Ley R, Liu Y-X, Loftfield E, Lozupone C, Maher M, Marotz C, Martin BD, McDonald D, McIver LJ, Melnik AV, Metcalf JL, Morgan SC, Morton JT, Naimey AT, Navas-Molina JA, Nothias LF, Orchanian SB, Pearson T, Peoples SL, Petras D, Preuss ML, Pruesse E, Rasmussen LB, Rivers A, Robeson MS, Rosenthal P, Segata N, Shaffer M, Shiffer A, Sinha R, Song SJ, Spear JR, Swafford AD, Thompson LR, Torres PJ, Trinh P, Tripathi A, Turnbaugh PJ, Ul-Hasan S, vander Hooft JJJ, Vargas F, Vázquez-Baeza Y, Vogtmann E, von Hippel M, Walters W, Wan Y, Wang M, Warren J, Weber KC, Williamson CHD, Willis AD, Xu ZZ, Zaneveld JR, Zhang Y, Zhu Q, Knight R, Caporaso JG (2019). Reproducible, interactive, scalable and extensible microbiome data science using QIIME 2. Nature Biotechnology.

[ref-4] Chen Y, Ma C, Liu L, He J, Zhu C, Zheng F, Dai W, Hong X, Liu D, Tang D, Dai Y (2021). Analysis of gut microbiota and metabolites in patients with rheumatoid arthritis and identification of potential biomarkers. Aging.

[ref-5] Chen J, Wright K, Davis JM, Jeraldo P, Marietta EV, Murray J, Nelson H, Matteson EL, Taneja V (2016). An expansion of rare lineage intestinal microbes characterizes rheumatoid arthritis. Genome Medicine.

[ref-6] Chen Y, Ye L, Zhu J, Chen L, Chen H, Sun Y, Rong Y, Zhang J (2024). Disrupted Tuzzerella abundance and impaired L-glutamine levels induce Treg accumulation in ovarian endometriosis: a comprehensive multi-omics analysis. Metabolomics.

[ref-7] Collaborators GBDRA (2023). Global, regional, and national burden of rheumatoid arthritis, 1990–2020, and projections to 2050: a systematic analysis of the Global Burden of Disease Study 2021. The Lancet Rheumatology.

[ref-8] Fieber C, Kovarik P (2014). Responses of innate immune cells to group A Streptococcus. Frontiers in Cellular and Infection Microbiology.

[ref-9] Fransen J, Van Riel PL (2009). The disease activity score and the EULAR response criteria. Rheumatic Disease Clinics of North America.

[ref-10] Frid P, Baraniya D, Halbig J, Rypdal V, Songstad NT, Rosen A, Berstad JR, Flato B, Alakwaa F, Gil EG, Cetrelli L, Chen T, Al-Hebshi NN, Nordal E, Al-Haroni M (2020). Salivary oral microbiome of children with juvenile idiopathic arthritis: a Norwegian cross-sectional study. Frontiers in Cellular and Infection Microbiology.

[ref-11] Hamdaoui N, Benkirane C, Bouaamali H, Azghar A, Mouncif M, Maleb A, Hammouti B, Al-Anazi KM, Kumar P, Yadav KK, Choi JR, Meziane M (2024). Investigating lactic acid bacteria genus Lactococcus lactis properties: antioxidant activity, antibiotic resistance, and antibacterial activity against multidrug-resistant bacteria Staphylococcus aureus. Heliyon.

[ref-12] Huang K, Gao X, Wu L, Yan B, Wang Z, Zhang X, Peng L, Yu J, Sun G, Yang Y (2021). Salivary microbiota for gastric cancer prediction: an exploratory study. Frontiers in Cellular and Infection Microbiology.

[ref-13] Kishikawa T, Maeda Y, Nii T, Motooka D, Matsumoto Y, Matsushita M, Matsuoka H, Yoshimura M, Kawada S, Teshigawara S, Oguro E, Okita Y, Kawamoto K, Higa S, Hirano T, Narazaki M, Ogata A, Saeki Y, Nakamura S, Inohara H, Kumanogoh A, Takeda K, Okada Y (2020). Metagenome-wide association study of gut microbiome revealed novel aetiology of rheumatoid arthritis in the Japanese population. Annals of the Rheumatic Diseases.

[ref-14] Klareskog L, Padyukov L, Ronnelid J, Alfredsson L (2006). Genes, environment and immunity in the development of rheumatoid arthritis. Current Opinion in Immunology.

[ref-15] Kononen E, Wade WG (2015). Actinomyces and related organisms in human infections. Clinical Microbiology Reviews.

[ref-16] Lee EH, Kim H, Koh JH, Cha KH, Lee KK, Kim WU, Pan CH, Lee YH (2022). Dysbiotic but nonpathogenic shift in the fecal mycobiota of patients with rheumatoid arthritis. Gut Microbes.

[ref-17] Lin H, Peddada SD (2024). Multigroup analysis of compositions of microbiomes with covariate adjustments and repeated measures. Nature Methods.

[ref-18] Liu X, Zou Q, Zeng B, Fang Y, Wei H (2013). Analysis of fecal *Lactobacillus* community structure in patients with early rheumatoid arthritis. Current Microbiology.

[ref-19] Maeda Y, Kurakawa T, Umemoto E, Motooka D, Ito Y, Gotoh K, Hirota K, Matsushita M, Furuta Y, Narazaki M, Sakaguchi N, Kayama H, Nakamura S, Iida T, Saeki Y, Kumanogoh A, Sakaguchi S, Takeda K (2016). Dysbiosis contributes to arthritis development *via* activation of autoreactive T cells in the intestine. Arthritis & Rheumatology.

[ref-20] Martin R, Rios-Covian D, Huillet E, Auger S, Khazaal S, Bermudez-Humaran LG, Sokol H, Chatel JM, Langella P (2023). Faecalibacterium: a bacterial genus with promising human health applications. FEMS Microbiology Reviews.

[ref-21] Minh Hoa TT, Darmawan J, Chen SL, Van Hung N, Thi Nhi C, Ngoc An T (2003). Prevalence of the rheumatic diseases in urban Vietnam: a WHO-ILAR COPCORD study. The Journal of Rheumatology.

[ref-22] Moentadj R, Wang Y, Bowerman K, Rehaume L, Nel H, Paraic OC, Stephens J, Baharom A, Maradana M, Lakis V, Morrison M, Wells T, Hugenholtz P, Benham H, Le Cao KA, Thomas R (2021). Streptococcus species enriched in the oral cavity of patients with RA are a source of peptidoglycan-polysaccharide polymers that can induce arthritis in mice. Annals of the Rheumatic Diseases.

[ref-23] Moon J, Lee AR, Kim H, Jhun J, Lee SY, Choi JW, Jeong Y, Park MS, Ji GE, Cho ML, Park SH (2023). Faecalibacterium prausnitzii alleviates inflammatory arthritis and regulates IL-17 production, short chain fatty acids, and the intestinal microbial flora in experimental mouse model for rheumatoid arthritis. Arthritis Research & Therapy.

[ref-24] Nhung PTT, Le HTT, Nguyen QH, Huyen DT, Quyen DV, Song LH, Van Thuan T, Tran TTT (2024). Identifying fecal microbiota signatures of colorectal cancer in a Vietnamese cohort. Frontiers in Microbiology.

[ref-25] Picchianti-Diamanti A, Panebianco C, Salemi S, Sorgi ML, Di Rosa R, Tropea A, Sgrulletti M, Salerno G, Terracciano F, D’Amelio R, Lagana B, Pazienza V (2018). Analysis of gut microbiota in rheumatoid arthritis patients: disease-related dysbiosis and modifications induced by etanercept. International Journal of Molecular Sciences.

[ref-26] Pradeepkiran JA (2019). Insights of rheumatoid arthritis risk factors and associations. Journal of Translational Autoimmunity.

[ref-27] Ridaura VK, Bouladoux N, Claesen J, Chen YE, Byrd AL, Constantinides MG, Merrill ED, Tamoutounour S, Fischbach MA, Belkaid Y (2018). Contextual control of skin immunity and inflammation by Corynebacterium. Journal of Experimental Medicine.

[ref-28] Rooney CM, Jeffery IB, Mankia K, Wilcox MH, Emery P (2024). Dynamics of the gut microbiome in individuals at risk of rheumatoid arthritis: a cross-sectional and longitudinal observational study. Annals of the Rheumatic Diseases.

[ref-29] Round JL, Lee SM, Li J, Tran G, Jabri B, Chatila TA, Mazmanian SK (2011). The Toll-like receptor 2 pathway establishes colonization by a commensal of the human microbiota. Science.

[ref-30] Scher JU, Abramson SB (2011). The microbiome and rheumatoid arthritis. Nature Reviews Rheumatology.

[ref-31] Scher JU, Sczesnak A, Longman RS, Segata N, Ubeda C, Bielski C, Rostron T, Cerundolo V, Pamer EG, Abramson SB, Huttenhower C, Littman DR (2013). Expansion of intestinal Prevotella copri correlates with enhanced susceptibility to arthritis. Elife.

[ref-32] Silman AJ, MacGregor AJ, Thomson W, Holligan S, Carthy D, Farhan A, Ollier WE (1993). Twin concordance rates for rheumatoid arthritis: results from a nationwide study. British Journal of Rheumatology.

[ref-33] Sun Y, Chen Q, Lin P, Xu R, He D, Ji W, Bian Y, Shen Y, Li Q, Liu C, Dong K, Tang YW, Pei Z, Yang L, Lu H, Guo X, Xiao L (2019). Characteristics of gut microbiota in patients with rheumatoid arthritis in Shanghai, China. Frontiers in Cellular and Infection Microbiology.

[ref-34] Tufail MA, Schmitz RA (2025). Exploring the probiotic potential of bacteroides spp. within one health paradigm. Probiotics and Antimicrobial Proteins.

[ref-35] Wang Q, Zhang SX, Chang MJ, Qiao J, Wang CH, Li XF, Yu Q, He PF (2022). Characteristics of the gut microbiome and its relationship with peripheral CD4(+) T cell subpopulations and cytokines in rheumatoid arthritis. Frontiers in Microbiology.

[ref-36] Wells G, Becker JC, Teng J, Dougados M, Schiff M, Smolen J, Aletaha D, van Riel PL (2009). Validation of the 28-joint Disease Activity Score (DAS28) and European League Against Rheumatism response criteria based on C-reactive protein against disease progression in patients with rheumatoid arthritis, and comparison with the DAS28 based on erythrocyte sedimentation rate. Annals of the Rheumatic Diseases.

[ref-37] Xu Z, Lv Z, Chen F, Zhang Y, Xu Z, Huo J, Liu W, Yu S, Tuersun A, Zhao J, Zong Y, Shen X, Feng W, Lu A (2022). Dysbiosis of human tumor microbiome and aberrant residence of Actinomyces in tumor-associated fibroblasts in young-onset colorectal cancer. Frontiers in Immunology.

[ref-38] Zhang X, Zhang D, Jia H, Feng Q, Wang D, Liang D, Wu X, Li J, Tang L, Li Y, Lan Z, Chen B, Li Y, Zhong H, Xie H, Jie Z, Chen W, Tang S, Xu X, Wang X, Cai X, Liu S, Xia Y, Li J, Qiao X, Al-Aama JY, Chen H, Wang L, Wu QJ, Zhang F, Zheng W, Li Y, Zhang M, Luo G, Xue W, Xiao L, Li J, Chen W, Xu X, Yin Y, Yang H, Wang J, Kristiansen K, Liu L, Li T, Huang Q, Li Y, Wang J (2015). The oral and gut microbiomes are perturbed in rheumatoid arthritis and partly normalized after treatment. Nature Medicine.

[ref-39] Zhao T, Wei Y, Zhu Y, Xie Z, Hai Q, Li Z, Qin D (2022). Gut microbiota and rheumatoid arthritis: from pathogenesis to novel therapeutic opportunities. Frontiers in Immunology.

[ref-40] Zhou B, Dong C, Zhao B, Lin K, Tian Y, Zhang R, Zhu L, Xu H, Yang L (2022). Bacteroides fragilis participates in the therapeutic effect of methotrexate on arthritis through metabolite regulation. Frontiers in Microbiology.

