# Supplementary material for: Preliminary insights into the gut microbiota of patients with rheumatoid arthritis in Vietnam
Source: PeerJ. 2025 Dec 18;13:e20521. doi: 10.7717/peerj.20521 (PMC12718522; doi:10.7717/peerj.20521)
Supplement: Supplemental Information 1 [file peerj-13-20521-s001.pdf]

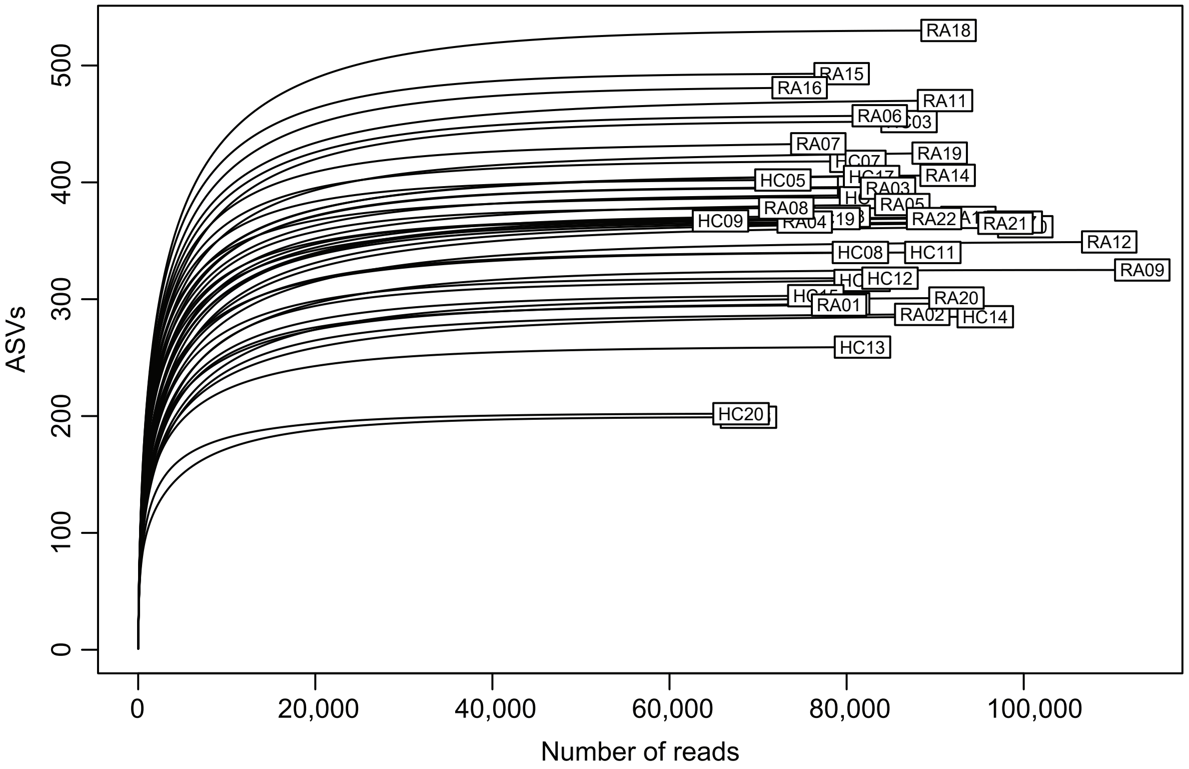


**Figure S1. Rarefaction curves were plotted from the amplicon sequence variant (ASV) table for each sample**. RA01-RA22, samples from patients with rheumatoid arthritis; HC01-HC20, samples from healthy controls.

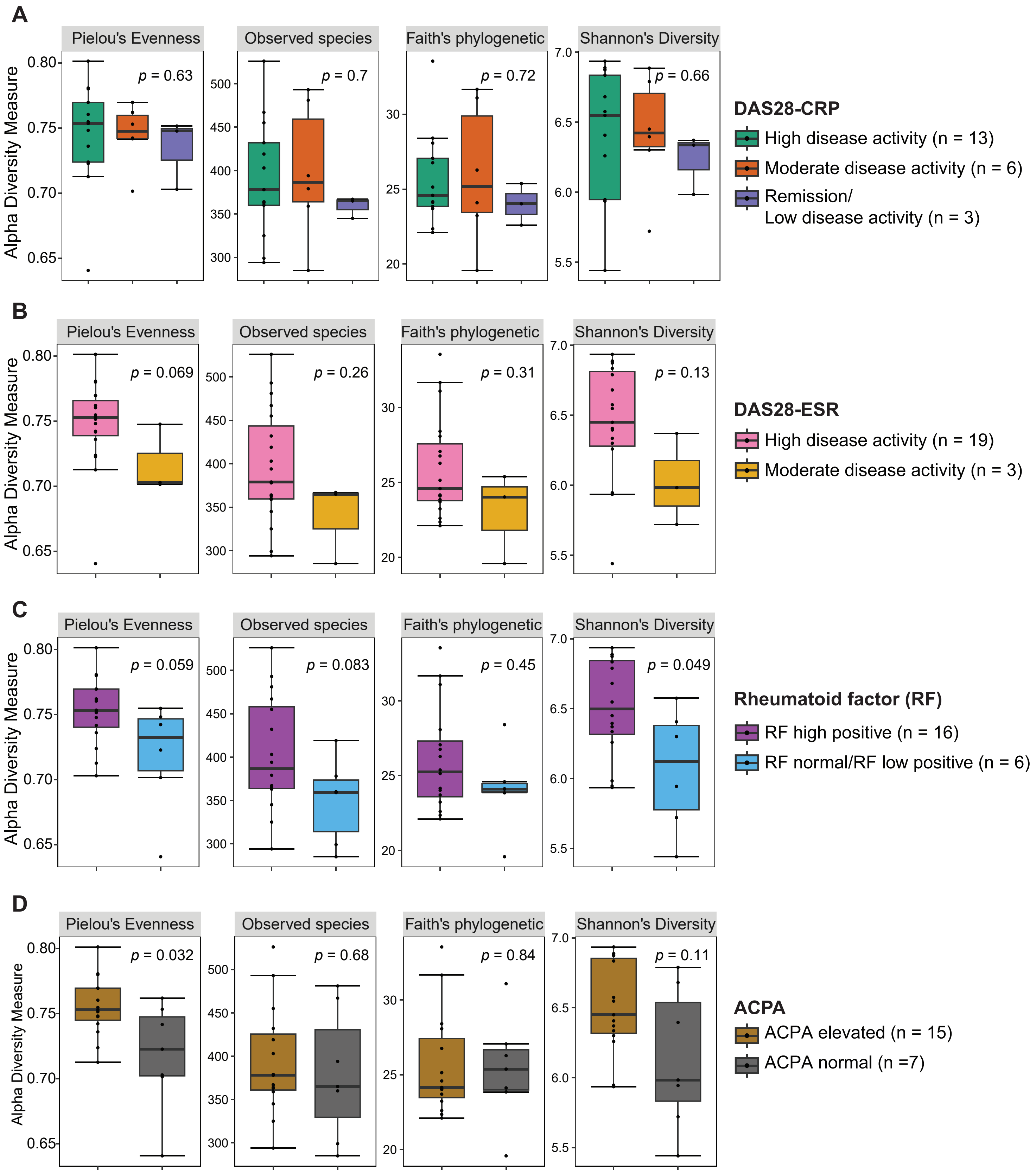


**Figure S2. Alpha diversity of gut microbiota in patients with rheumatoid arthritis grouped using clinical indicators.** **A**) Disease activity (high, moderate, low/remission) in function of the DAS28-CRP score. **B**) Disease activity (high, moderate) in function of the DAS28-ESR score. **C**) Concentration (high versus low/normal) of rheumatoid factor and **D**) of Anti-citrullinated protein antibodies (ACPA). *P* values were calculated with the Kruskal–Wallis test for the three DAS28-ESR subgroups, and with the Mann–Whitney U test for the other clinical indicators.

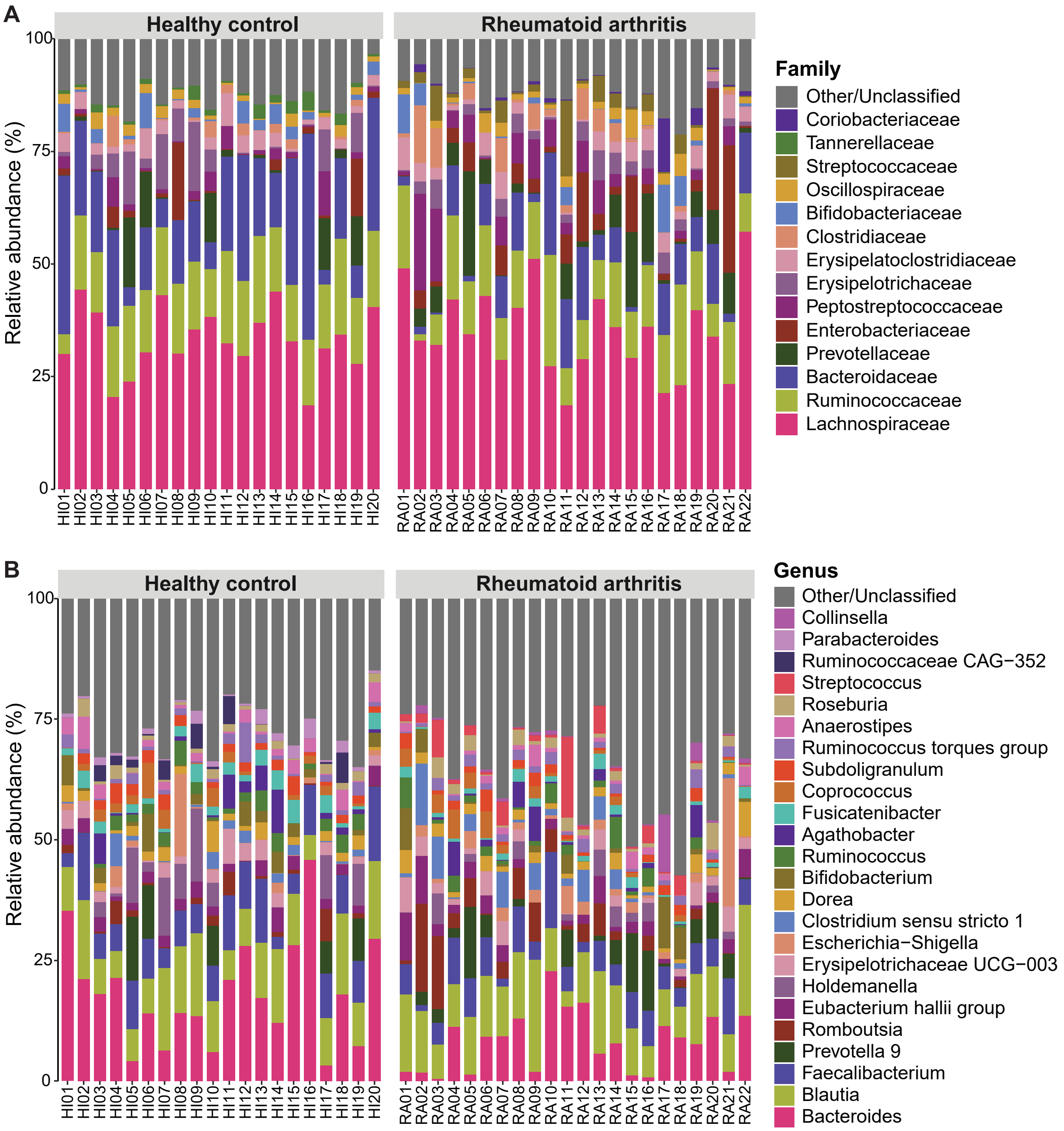


**Figure S3. Gut microbial communities at the family (A) and genus (B) levels in patients with rheumatoid arthritis and healthy controls.** Other/Unclassified, families/genera representing <1% of the relative abundance and unclassified taxa. HI, healthy control; RA, patient with rheumatoid arthritis.

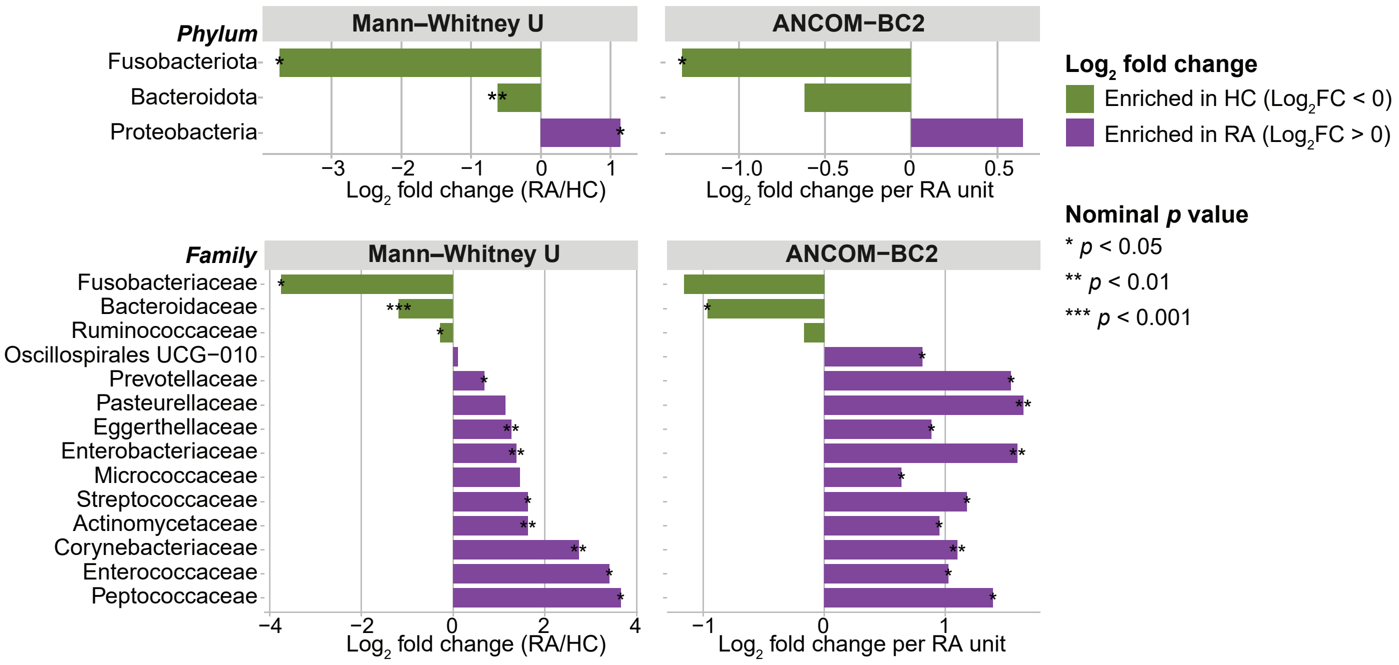


**Figure S4.** Phyla and families with significantly different abundances between patients with rheumatoid arthritis (RA) and healthy controls (HC) using either the Mann–Whitney U test or ANCOM-BC2 (nominal *p* values < 0.05). Violet, taxa enriched in the RA group; green, taxa enriched in the HC group.
